# Complementary Error Patterns Between Human Evaluators and GPT-4o in Video-Based Cardiopulmonary Resuscitation Skills Assessment: Implications for Artificial Intelligence-Assisted Second Reading

**DOI:** 10.3390/jcm15124436

**Published:** 2026-06-08

**Authors:** Hye Ji Park, Daun Choi, Choung Ah Lee

**Affiliations:** 1 Department of Emergency Medicine, Hallym University Dongtan Sacred Heart Hospital, Hwaseong 18450, Republic of Korea; ji4051@hanmail.net; 2 Hallym Dongtan Simulation Center, 160, Samsung 1-ro, Hwaseong 18450, Republic of Korea; ggch04650@naver.com; 3 Research Institute for Complementary and Alternative Medicine, Hallym University, Chuncheon 24342, Republic of Korea

**Keywords:** cardiopulmonary resuscitation, artificial intelligence, GPT-4o, vision-language model, skills assessment, medical education

## Abstract

**Background/Objectives**: Cardiopulmonary resuscitation (CPR) skill assessments are susceptible to evaluator subjectivity, cognitive fatigue, and observational limitations. Although recent advances in multimodal artificial intelligence have increased the possibility of automated video-based assessment, its validity for clinical skill evaluation remains insufficiently examined. **Methods:** In this cross-sectional study, we enrolled 130 laypersons who underwent Basic Life Support training and skill testing. Twenty recordings were used for prompt development and 110 recordings were analyzed. Expert evaluators and GPT-4o independently assessed participants’ skills using a 12-item checklist. The manikin sensor data were the reference standard for the four chest compression metrics. Agreement was evaluated using Gwet’s agreement coefficient 1 (AC1) and intraclass correlation coefficient (2,1). Diagnostic accuracy, sensitivity, and specificity were compared using McNemar’s test. **Results**: Procedural items such as confirming cardiac arrest, calling 119, and requesting an automated external defibrillator showed a near-perfect agreement between experts and GPT-4o (AC1 > 0.8). However, the agreement was poor for the compression depth (AC1 = 0.374) and full chest recoil (AC1 = 0.355). Experts demonstrated high sensitivity (77.8–84.3%) but low specificity (24.6–47.8%), whereas GPT-4o showed low sensitivity (35.6–40.6%) but high specificity (69.2–76.1%). **Conclusions**: GPT-4o cannot serve as a standalone evaluator because of its inherent limitations in inferring three-dimensional spatial information from two-dimensional videos. However, its high agreement on procedural items and complementary error patterns with that of human evaluators on compression metrics suggests its potential as a decision support tool to mitigate expert leniency bias in CPR education.

## 1. Introduction

Out-of-hospital cardiac arrest remains a leading cause of death worldwide, and early bystander cardiopulmonary resuscitation (CPR) is among the most critical determinants of survival [1,2]. Accordingly, CPR education and the reliable assessment of acquired skills are of paramount importance [2,3]. However, evaluation of CPR skills is challenging. Variability in expert judgment regarding chest compression depth and rate and the subjectivity involved in observing chest rise during rescue breaths are major sources of assessment inconsistencies [4]. Furthermore, the repetitive nature of CPR performance extends examination duration, leading to evaluator fatigue and diminished concentration, which may negatively affect the assessment [4,5,6]. These limitations highlight the need for novel assessment tools that can objectively and consistently evaluate CPR skills.

Artificial intelligence (AI) technologies have been established to complement human intuition and judgment across diverse domains. Specifically, GPT-4o, a multimodal large language model with integrated vision capabilities, can process both textual and visual information and draw logical conclusions from learned patterns [7,8]. Its potential as a clinical decision-support tool and educational adjunct has been increasingly reported in medical literature. The application of GPT-4o to CPR assessment could enable a standardized evaluation of training quality and provide individualized feedback, enhancing educational effectiveness [9]. However, concerns regarding the output stability of large language models and limited empirical evidence regarding their use in high-stake assessment environments have led medical professionals to take a cautious stance [10,11,12].

Previous studies demonstrated the potential of AI in skill-based medical education. Some investigations have shown that AI can achieve a certain level of performance in evaluating CPR skills [9,13], whereas others have demonstrated its ability as a data-driven analytical tool [13,14]. However, findings regarding the use of GPT-4o have been inconsistent across studies [10,12]. Most studies were confined to single-dimensional assessments or specific scenarios and lacked the multidimensional analysis required in real-world educational settings. Existing research has predominantly focused on AI as an examinee (i.e., answering examination questions) rather than as an evaluator of clinical performance [10,11,12]. Moreover, most studies have relied on text-based inputs or text–image combinations, leaving the reliability of AI in analyzing video recordings of actual clinical skill performance insufficiently validated. Notably, no prior study has examined whether a general-purpose VLM can function as an independent evaluator—rather than an examinee—of video-recorded CPR performance, benchmarked simultaneously against certified human evaluators and objective sensor-derived data.

In this study, we aimed to compare the assessment of CPR skills in laypersons (individuals without professional healthcare training) who underwent BLS training, as evaluated by expert instructors (on-site and video-based) and GPT-4o. Using manikin sensor data as the reference standard, we sought to characterize the assessment capabilities of human and AI evaluators and investigate the potential role of multimodal AI as an assessment tool in CPR education.

## 2. Materials and Methods

### 2.1. Study Design and Participants

This cross-sectional study was designed to evaluate the potential of GPT-4o (OpenAI, San Francisco, CA, USA) as an evaluator of practical CPR skills. We enrolled adults aged ≥18 who met the following inclusion criteria: (1) no professional healthcare license or active healthcare role and (2) willingness to provide written informed consent and to be video-recorded. Exclusion criteria were: (1) holders of a medical, nursing, paramedic, or allied-health license; (2) current BLS provider or instructor certification; and (3) physical conditions precluding chest compressions. The target sample size of 110 analyzable recordings was determined based on the primary outcome of inter-rater agreement using Gwet’s agreement coefficient 1 (AC1) for dichotomous items [15]. Following the precision-based framework described by Gwet, this sample size yields confidence interval half-widths of approximately ±0.09 for expected AC1 values of 0.70–0.80 with binary outcomes. To account for potential dropouts and the allocation of 20 recordings for prompt development, 130 participants were enrolled. Between 1 January and 30 November 2025, individuals who registered for CPR training at the Simulation Center of Hallym University Dongtan Sacred Heart Hospital were recruited. Written informed consent was obtained from all participants and instructors before enrollment. This study was approved by our Institutional Review Board of Hallym University Dongtan Sacred Heart Hospital (HDT 2024-11-014).

### 2.2. CPR Training and Assessment Procedure

All participants underwent BLS training and subsequently performed a CPR skill test on a sensor-equipped manikin following the American Heart Association (AHA) BLS guidelines. The assessment checklist comprises 12 items across four domains: initial patient assessment, chest compressions, repeated performance cycles, and defibrillation. The checklist excluded pulse checks and rescue breathing. Each item is scored dichotomously, 1 (performed/adequate) or 0 (not performed/inadequate). The detailed scoring criteria are presented in Appendix A.

**Figure 1:**
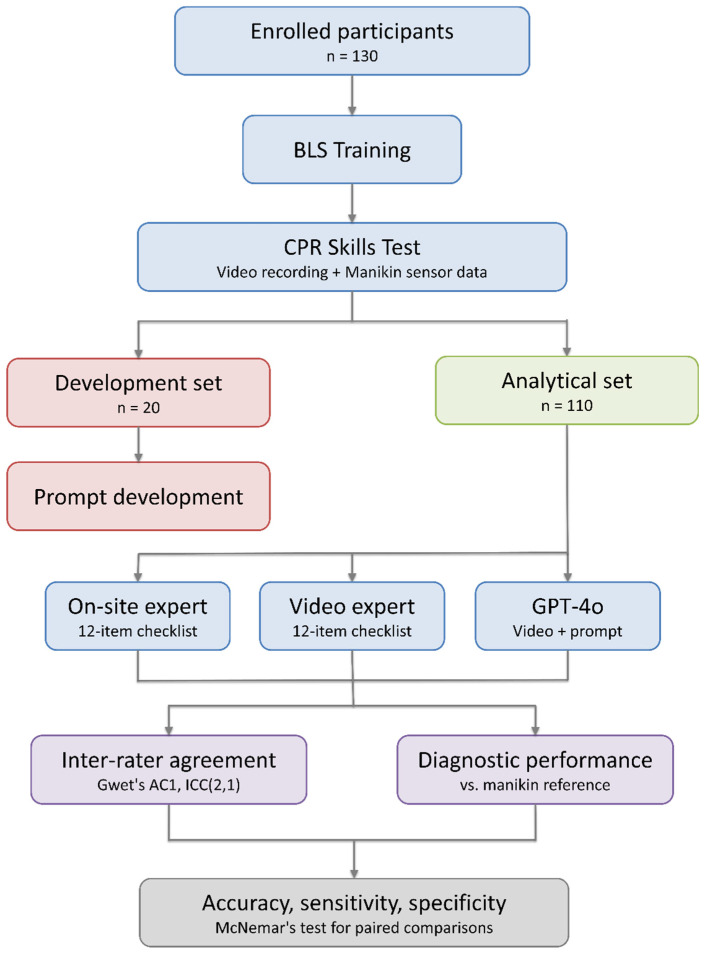
Study design and evaluation framework.

Skill tests were video-recorded from two perspectives: a lateral view of the manikin (equivalent to a frontal view of the rescuer), with cameras positioned at the rescuer’s elbow height and oriented horizontally parallel to the ground plane. This setup was designed to capture the vertical axis of chest displacement within the camera’s imaging plane. To protect the participants’ privacy, facial regions were excluded from the camera’s field of view. Quantitative chest compression performance data (hand position, compression rate, compression depth, and full chest recoil) were automatically recorded by the built-in sensors of the manikin and served as the reference standard for evaluating evaluator accuracy. The overall flowchart of this study is shown in Figure 1.

### 2.3. Expert Evaluation

Expert evaluations were performed by 7 certified AHA BLS instructors with a mean of 3 years of BLS instruction experience. Prior to formal data collection, all evaluators participated in a calibration exercise using the 20-video development set, during which the operational definition of each checklist item was discussed and scoring discrepancies were resolved by consensus. The development set was then excluded from all subsequent analyses. On-site evaluation was performed immediately after each participant’s skill test using the standardized checklist. The expert evaluators were blinded to the manikin sensor data and GPT-4o evaluation results at all times. Subsequently, the same instructors independently reviewed the recorded videos after a wash-out interval of 2 weeks and reevaluated performance using an identical checklist (video-based evaluation). Each item was scored as 1 (clearly observed and adequate) or 0 (not observed or inadequate).

### 2.4. AI-Based Evaluation Using GPT-4o

All AI evaluations were conducted using GPT-4o (OpenAI, San Francisco, CA, USA), which was accessed through the official ChatGPT web interface. The specific model version available during the study period was GPT-4o, as deployed through the ChatGPT Plus subscription between 1 January 2025, and 30 November 2025. The web interface was selected for its native video upload capability; however, it should be noted that the ChatGPT web platform undergoes continuous backend updates by OpenAI, which may limit strict reproducibility. Importantly, the web interface does not expose user-controllable hyperparameters such as temperature, top-p, or system seed; these settings could not therefore be standardized across evaluations. The exact model checkpoint and internal video processing parameters (e.g., frame sampling rate and spatial resolution of extracted frames) are not publicly disclosed by OpenAI, and therefore cannot be controlled or reported with precision.

A structured zero-shot prompt was developed that included the complete AHA BLS assessment criteria for all 12 checklist items, explicit scoring definitions (1 = clearly observed and adequate; 0 = not observed or inadequate), and instructions to provide a written justification for each assigned score. Prompt development was conducted iteratively using 20 randomly selected videos (the development set), with refinements made to minimize ambiguous scoring outputs and improve adherence to the assessment rubric. During refinement, the scoring principles were further reinforced to ensure a strict and conservative evaluation, and all assessments were constrained to be based solely on observable evidence within the video. This iterative refinement process was continued until the evaluation outputs consistently satisfied the predefined criteria and the variability in interpretation was minimized. The final prompt was then fixed and applied consistently without further modification in all subsequent analyses. The final prompts are shown in Table S1.

For the analytical phase, each of the remaining 110 videos was analyzed in a separate independent ChatGPT session to prevent carryover effects from prior evaluations. The video file was uploaded directly to the ChatGPT interface, and the structured prompt was entered in the same session. The GPT-4o returned scores for all 12 items, along with text-based justifications. When the model produced technical errors (e.g., failure to process the video) or misapplied the scoring rubric (e.g., scoring an item not depicted in the video), the same video was reanalyzed in a new session. A total of 11 videos required re-analysis: 2 due to technical processing failures (e.g., the model failing to load the video) and 9 due to clear misapplication of the scoring rubric. The frequency and nature of the re-analyses were recorded.

### 2.5. Technical Considerations of Video Analysis by Vision–Language Models

Several inherent technical limitations of the current vision–language models (VLMs) in processing video data are relevant for interpreting the present results and are summarized here to provide methodological transparency.

First, VLMs, such as GPT-4o, do not natively process videos as a continuous temporal stream. Instead, the system extracts a limited number of still frames from the uploaded video and analyses them as sequences of static images [16]. The frame sampling rate employed by the ChatGPT platform is not publicly documented but is generally estimated at 1–2 frames per second (FPS) based on available technical analyses [16,17]. CPR chest compressions occur at a guideline-recommended rate of 100–120 compressions per minute, corresponding to a frequency of approximately 1.67–2.0 Hz. According to the Nyquist–Shannon sampling theorem, accurately capturing the peak (maximum depth) and trough (full recoil) of such periodic motions requires a sampling rate of at least twice the signal frequency [18]. Therefore, a sampling rate of 1–2 FPS is insufficient to capture the critical extremes of each compression cycle, resulting in systematic information loss regarding compression depth and chest recoil quality [19].

Secondly, inferring three-dimensional (3D) spatial depth from two-dimensional (2D) monocular videos is a well-recognized challenge in computer vision [20]. Chest compression depth involves a vertical displacement of 5–6 cm. Although the cameras were positioned laterally to the manikin at elbow height with horizontal orientation to align the compression axis with the imaging plane, no extrinsic camera calibration or known physical reference scale was present within the field of view, precluding pixel-displacement-to-millimeter conversion. Combined with occlusion by the rescuer’s hands and arms and motion blur inherent to rapid repetitive movements, the visual evidence available to the model for quantitative depth estimation remains severely constrained even under these geometrically favorable conditions. These factors collectively explain why the VLM performance is expected to be substantially lower for compression depth and recoil assessment than for procedural items involving discrete, clearly visible actions.

### 2.6. Statistical Analysis

Agreement between evaluators on the 12 dichotomous checklist items was assessed using Gwet’s AC1 statistic, which is robust to the kappa paradox that arises when category prevalence is highly skewed [21]. The 95% confidence intervals (CIs) for AC1 were computed using the CAC package (version 1.0). Agreement on total scores (continuous variable, range 0–12) was evaluated using the intraclass correlation coefficient (ICC) under a two-way random-effects model with single measures and consistency (ICC(2,1)), computed using the irrCAC package (version 0.84.1). AC1 values were interpreted according to Landis and Koch [22]: <0.20, poor; 0.21–0.40, fair; 0.41–0.60, moderate; 0.61–0.80, substantial; and 0.81–1.00, almost perfect. The ICC values were interpreted according to Koo and Li [23]: <0.50, poor; 0.50–0.75, moderate; 0.75–0.90, good; and > 0.90, excellent.

The diagnostic performance (accuracy, sensitivity, and specificity) of each evaluator was calculated against that of the manikin sensor which was used as the reference standard for the four chest compression metrics using the caret package (version 6.0-94). Paired comparisons of accuracy between the evaluators were performed using McNemar’s test for correctness vectors with 95% Wald CIs. Sensitivity and specificity comparisons were performed using DTComPair software (version 1.0.3). Statistical significance was defined as a two-sided *p*-value of < 0.05. All analyses were performed using the R software version 4.5.1 (R Foundation for Statistical Computing, Vienna, Austria).

## 3. Results

### 3.1. Dataset Composition

Overall, 130 individuals participated in the BLS training program, yielding 130 CPR skill test recordings. Of these, 20 were allocated to the GPT-4o prompt development (development set), and the remaining 110 recordings constituted the analytical dataset. The baseline characteristics of the participants are presented in Table 1. The mean age of participants was 22.99 ± 2.03 years, and 30.9% of the participants were male. Most participants had received CPR training at least once.

### 3.2. Scoring Results

The mean (standard deviation [SD]) total scores for expert on-site evaluation, expert video-based evaluation, and GPT-4o evaluation were 9.63 (2.12), 9.48 (1.98), and 9.39 (1.91), respectively. The domain-level scores are presented in Table 2. For all evaluators, the patient assessment items received the highest scores, whereas the chest compression items showed the greatest variability.

### 3.3. Inter-Evaluator Agreement

Agreement analyses across the 12 dichotomous items using Gwet’s AC1 are presented in Table 3. When examining the three-way agreement among all evaluators (on-site expert, video expert, and GPT-4o), items demonstrating almost perfect agreement (AC1 > 0.80) included cardiac arrest response confirmation (0.994), AED power on (0.940), hand position/posture (0.889), calling 119 (0.847), and requesting an AED (0.844). Moderate to substantial agreement (AC1 0.50–0.80) was observed for: pad placement (0.775), compression rate (0.718), breathing check (0.650), standing clear for shock delivery (0.614), and standing clear for rhythm analysis (0.545). Poor agreement (AC1 < 0.40) was observed for compression depth (0.374) and full chest recoil (0.355). The ICC(2,1) for the total score among all three evaluators was 0.769 (moderate).

### 3.4. Diagnostic Performance Against Manikin Reference Standard

Table 4 presents the accuracy, sensitivity, and specificity of each evaluator against those of the manikin sensor for the four chest compression items. Expert on-site evaluations demonstrated a consistent pattern of high sensitivity (77.8–84.3%) coupled with low specificity (24.6–47.8%), indicating a systematic tendency to rate inadequate performance as adequate (leniency bias). The expert video-based evaluation exhibited a similar pattern. In contrast, GPT-4o displayed the opposite pattern for compression depth and full chest recoil: low sensitivity (35.6–40.6%), but high specificity (69.2–76.1%), indicating a tendency to rate adequate performance as inadequate (conservative bias). For hand position, GPT-4o achieved very high sensitivity (94.0%) but extremely low specificity (11.1%), suggesting near-universal “adequate” ratings regardless of actual performance.

### 3.5. Paired Comparisons of Diagnostic Performance

McNemar’s test results comparing the expert and GPT-4o evaluation accuracies are presented in Table 5. For the compression rate, the expert on-site evaluation was significantly more accurate than that of GPT-4o (+16.4%, *p* = 0.002), with a significantly higher specificity (+25.0%, *p* < 0.001). For compression depth, experts showed significantly higher sensitivity (+40.6%, *p* < 0.001) while GPT-4o demonstrated significantly higher specificity (−28.3%, *p* = 0.004). Full chest recoil showed the most pronounced divergence; for experts, sensitivity was significantly higher (+42.2%, *p* < 0.001), whereas for GPT-4o specificity was significantly higher (−44.6%, *p* < 0.001). No significant difference in accuracy was observed for hand position (*p* = 0.386). Similar patterns were observed when comparing expert video-based evaluations with those of GPT-4o.

## 4. Discussion

To our knowledge, this study provides the first comprehensive evaluation of a multimodal VLM (GPT-4o) as an assessor of practical CPR skills using video analysis benchmarked against both expert human evaluators and objective manikin sensor data. While previous studies primarily evaluated AI-based CPR assessment in comparison with a single human evaluator [13], the present study employed a triangulated evaluation framework incorporating on-site expert evaluation, video-based expert evaluation, and AI-based evaluation. Additionally, an objective reference standard derived from the manikin sensor data was used to validate the assessment accuracy. The present work makes three contributions to the literature: (1) the first benchmarking of a general-purpose multimodal VLM as a CPR skills evaluator against both certified human evaluators and objective sensor data simultaneously; (2) the identification of diametrically opposing error patterns—human leniency versus AI conservatism—across psychomotor compression metrics; and (3) the proposal of a hybrid AI-as-second-reader assessment paradigm for CPR education, analogous to established AI double-reading workflows in diagnostic radiology. Our findings revealed a pattern of systematically opposing error profiles between human and AI evaluators, which has important implications for the future integration of AI into medical skills education.

### 4.1. Complementary Error Patterns: Human Leniency vs. AI Conservatism

The most striking finding of this study was the diametrically opposing bias patterns between human experts and GPT-4o when evaluated in comparison with the manikin as a reference standard. Expert evaluators consistently demonstrated high sensitivity but low specificity across chest compression metrics, reflecting a leniency bias, a well-documented phenomenon in performance-based assessment wherein evaluators tend to give learners the benefit of doubt [4,6]. This pattern was consistent across both the on-site and video-based expert evaluations, suggesting that leniency bias is an intrinsic characteristic of human observation rather than an artifact of the evaluation setting.

Conversely, GPT-4o exhibited high specificity but low sensitivity for compression depth and full chest recoil, indicating a conservative judgment pattern. If the model did not identify clear visual evidence confirming adequate performance, it defaulted to an “inadequate” rating. This behavior is consistent with the “better-safe-than-sorry” overreaction pattern described in recent VLM research [24,25]. From an educational safety perspective, this conservatism has a paradoxical advantage. While it may underrate competent learners, it is less likely to falsely validate learners whose technique is genuinely deficient, which is a clinically dangerous error that human evaluators commit with considerable frequency, as our data demonstrate.

### 4.2. Technical Limitations of VLMs in Dynamic Skills Assessment

The poor agreement and low sensitivity of GPT-4o for compression depth and full chest recoil can be explained by two fundamental technical constraints of the current VLMs applied to video analysis.

First, the temporal-resolution limitation arising from frame subsampling is critical. As described in Section 2, GPT-4o processes videos by extracting a limited number of still frames, typically at 1–2 FPS. At the guideline-recommended compression rate of 100–120/min (1.67–2.0 Hz), this sampling rate violates the Nyquist–Shannon criterion, which requires a minimum of twice the signal frequency to faithfully reconstruct the waveform [18,19]. Consequently, the precise moments are unlikely to be captured in any given extracted frame, depriving the model of the critical visual evidence needed to accurately assess these parameters.

Second, the inherent difficulty of inferring 3D spatial depth from 2D monocular images presents a fundamental challenge [20]. The compression depth involves a physical displacement of approximately 5–6 cm along an axis nearly perpendicular to the camera plane. This displacement provides insufficient pixel-level information for reliable quantitative depth estimation. Notably, the cameras were positioned to maximize visibility of vertical chest displacement; the persistent failure of GPT-4o to estimate compression depth accurately under these favorable conditions highlights that this limitation is intrinsic to current VLM architecture rather than attributable to suboptimal recording geometry. Unlike specialized computer vision systems that employ depth-sensing cameras or pose estimation algorithms trained specifically for CPR assessment [19,26], general-purpose VLMs lack the architectural features necessary for precise 3D spatial reasoning from single-viewpoint videos.

In contrast, procedural items, such as confirming the cardiac arrest response, calling 119, or requesting an AED, achieved near-perfect agreement between GPT-4o and human evaluators (AC1 > 0.80). These items involve discrete, temporally sustained actions with clear visual markers (e.g., the presence or absence of a specific gesture or vocalization) that can be reliably identified from a small number of still frames. This finding aligns with the broader VLM literature demonstrating strong performance in binary presence–absence tasks but limited capability in quantifying continuous physical parameters [16].

### 4.3. The Exceptional Case of Compression Rate

Compression rate evaluation using GPT-4o requires special attention. Despite rate being a temporal parameter calculable in principle from frame sequences, GPT-4o demonstrated the lowest specificity among all items (4.7%), essentially rating nearly all performers as “adequate.” This paradoxical finding likely reflects the interaction between the low frame sampling rate and the high frequency of compression motion. At 1–2 FPS, the model cannot reliably count individual compressions within a given time interval and may instead rely on heuristic visual cues (e.g., the general appearance of rhythmic motion) rather than precise temporal measurements. Expert evaluators who could perceive rhythm in real time significantly outperformed GPT-4o in this metric (accuracy: 51.8% vs. 35.5%, *p* = 0.002). Therefore, the current VLMs should not be relied upon for any assessment parameters that require precise temporal quantification from videos.

### 4.4. Implications for CPR Education: Toward a Hybrid Assessment Model

Our findings do not support the use of GPT-4o as a standalone independent evaluator for CPR skill assessment. The low sensitivity for compression depth and recoil and near-complete failure in rate specificity render unsupervised AI-only assessments clinically unacceptable. However, dismissing AI entirely would be an equally erroneous conclusion.

The complementary error patterns observed in this study suggest a potential hybrid assessment model in which AI functions as a “second reader” or decision-support tool alongside human evaluators. Specifically, GPT-4o’s high specificity for compression depth (76.1%) and recoil (69.2%) could serve as a check against the well-documented leniency bias of human evaluators who demonstrated a specificity of only 47.8% and 24.6%, respectively, for these same parameters. In such a model, cases where the human evaluator rates performance as “adequate” but the AI flags it as “inadequate” would automatically be routed for secondary review. This approach mirrors established practices in diagnostic radiology, where AI-assisted double reading has improved detection rates without replacing the radiologist’s judgment [27,28,29].

Furthermore, the near-perfect AI–human agreement on procedural items suggests that these components could be reliably delegated to AI-based automated assessment, freeing human evaluators from focusing their cognitive resources on the more complex psychomotor aspects of compression quality that currently exceed AI capabilities. This selective automation strategy could reduce evaluator cognitive load and fatigue, factors that our study suggests contribute to leniency bias, while maintaining the human oversight necessary for patient-safety-critical parameters. In practice, such a hybrid model would enable AI to automatically score procedural items from video recordings, flagging only compression-quality concerns for focused expert review. Integration with API-based automated pipelines, rather than manual web-interface interactions as employed in this study, would enable scalable, real-time deployment in institutional CPR training programs.

### 4.5. Limitations

This study has some limitations. First, the use of the ChatGPT web interface rather than the GPT-4o API with controlled hyperparameters (e.g., temperature = 0) limits the reproducibility of our findings because OpenAI continuously updates the web platform’s backend model weights and safety filters. In addition, each video was evaluated only once by GPT-4o, and intra-model variability across repeated runs was not assessed. Future studies should perform replicate evaluations to quantify the stochastic variability inherent to large language model outputs, ideally using the API with fixed model versions and documented parameters. Second, the exact frame sampling rate and spatial resolution used by the ChatGPT platform for video processing are proprietary and cannot be reported, precluding precise characterization of the temporal information available to the model. Third, our analysis did not include a systematic qualitative error analysis of GPT-4o’s text-based justifications, which would provide deeper insight into whether scoring errors arose from visual hallucinations, spatial confusion, or incorrect medical reasoning. Such analysis will provide an important direction for future studies. Fourth, because the same expert instructors performed both on-site and subsequent video-based evaluations, recall bias may have inflated the agreement between the two modalities. Future studies should employ independent evaluators for on-site and video-based assessments to minimize this potential confounding factor. Fifth, this study was conducted at a single institution with a homogeneous participant population (laypersons), which may limit its generalizability to other trainee populations. Sixth, the study did not adhere to specific AI reporting guidelines such as DECIDE-AI or STARD-AI; however, the essential elements recommended by these frameworks, including reference standard specification, model identification, and error characterization, have been addressed where possible. Seventh, this study evaluated a single general-purpose VLM. Purpose-built computer vision systems incorporating pose estimation or depth-sensing technologies may yield substantially different performance profiles and should be evaluated in future comparative studies.

## 5. Conclusions

This study demonstrates that GPT-4o can reliably assess procedural components of CPR performance from video with near-perfect agreement with human experts, but performs poorly on dynamic chest compression quality parameters because of the inherent limitations of current vision–language models in temporal resolution and 3D spatial inference from 2D video. The systematically opposing error patterns between human evaluators (leniency bias) and GPT-4o (conservative bias) suggest that AI is best positioned not as a replacement for human assessment but as a complementary second reader capable of flagging potentially lenient human ratings for review. Future research should focus on purpose-built video analysis systems with adequate temporal resolution, systematic qualitative error taxonomies, and prospective validation of hybrid human–AI assessment models in CPR education settings.

## Figures and Tables

**Table 1:** Baseline characteristics of the participants.

Variable	Total (n = 110)
Age (years)	22.99 ± 2.03 years
Sex	
Male, ***n*** (%)	34 (30.9%)
Female, ***n*** (%)	76 (69.1%)
Previous CPR training	
None, ***n*** (%)	48 (43.6%)
Once, ***n*** (%)	62 (56.4%)

**Table 2:** Grading results of patient assessment, chest compressions, Defibrillation, and total score for the participants, as reported by the experts and GPT-4o.

	Grading Scores	Patient Assessment	Chest Compression	Defibrillation	Total
Expert (on-site)	Mean (SD)	3.45 (0.86)	3.03 (1.04)	3.15 (0.96)	9.63 (2.12)
	Median (IQR)	4 (3, 4)	3 (2, 4)	3 (2, 4)	10 (8, 11)
Expert (Video)	Mean (SD)	3.47 (0.84)	2.90 (0.98)	3.11 (0.87)	9.48(1.98)
	Median (IQR)	4 (3, 4)	3 (2, 4)	3 (3, 4)	10 (8, 11)
GPT-4o	Mean (SD)	3.58 (0.88)	2.47 (1.12)	3.34 (0.55)	9.39 (1.91)
	Median (IQR)	4 (4, 4)	2 (2, 4)	3 (3, 4)	10 (8, 11)

SD: standard deviation, IQR: interquartile range.

**Table 3:** Inter-rater agreement of CPR performance items among on-site experts, video evaluators, and GPT-4o using Gwet’s AC1.

Item	On-Site vs. Video Evaluators		On-Site vs. GPT-4o Evaluators		Video vs. GPT-4o Evaluators		Among All Three Evaluators	
	Agreement (%)	AC1 (95% CI)	Agreement (%)	AC1 (95% CI)	Agreement (%)	AC1 (95% CI)	Agreement (%)	AC1 (95% CI)
Cardiac arrest response	100.0	1.00 (1.00–1.00)	99.1	0.99 (0.97–1.00)	99.1	0.99 (0.97–1.00)	99.1	0.99 (0.98–1.00)
Call 119	94.5	0.94 (0.88–0.99)	81.8	0.77 (0.66–0.88)	87.3	0.83 (0.74–0.93)	81.8	0.85 (0.78–0.92)
Request AED	94.5	0.93 (0.87–0.99)	81.8	0.77 (0.66–0.88)	87.3	0.83 (0.74–0.93)	81.8	0.84 (0.77–0.92)
Check breathing	90.9	0.84 (0.75–0.94)	66.4	0.51 (0.34–0.68)	73.6	0.64 (0.49–0.78)	65.5	0.65 (0.53–0.77)
Hand position/posture	97.3	0.96 (0.92–1.00)	89.1	0.86 (0.78–0.94)	88.2	0.85 (0.77–0.94)	87.3	0.89 (0.83–0.95)
Compression rate	93.6	0.90 (0.83–0.98)	72.7	0.61 (0.46–0.76)	75.5	0.66 (0.52–0.80)	70.9	0.72 (0.61–0.82)
Compression depth	84.5	0.71 (0.58–0.84)	53.6	0.07 (0.00–0.26)	67.3	0.35 (0.17–0.53)	52.7	0.374 (0.25–0.50)
Full chest recoil	91.8	0.86 (0.77–0.95)	50.9	0.03 (0.00–0.22)	57.3	0.15 (0.00–0.33)	50.0	0.36 (0.23–0.48)
AED power on	97.3	0.97 (0.93–1.00)	91.8	0.91 (0.85–0.97)	94.5	0.941 (0.892–0.990)	91.8	0.94 (0.89–0.98)
Pad placement	85.5	0.81 (0.71–0.91)	76.4	0.68 (0.54–0.81)	89.1	0.85 (0.75–0.94)	75.5	0.78 (0.68–0.87)
Stand clear for analysis	80.9	0.65 (0.50–0.80)	65.5	0.37 (0.18–0.56)	80.9	0.63 (0.48–0.78)	63.6	0.55 (0.42–0.67)
Stand clear for shock	85.5	0.75 (0.62–0.87)	61.8	0.43 (0.25–0.61)	76.4	0.69 (0.56–0.82)	61.8	0.61 (0.49–0.74)
Total score	-	0.88 (0.83–0.92) *	-	0.68 (0.56–0.77) *	-	0.74 (0.64–0.81) *	-	0.77 (0.70–0.83) *

AC1 values were interpreted as follows: <0.20, poor; 0.21–0.40, fair; 0.41–0.60, moderate; 0.61–0.80, substantial; 0.81–1.00, almost perfect. * Total score ICC values were interpreted as follows: <0.50, poor; 0.50–0.75, moderate; 0.75–0.90, good; >0.90, excellent AC1, Gwet’s agreement coefficient 1; AED, automated external defibrillator; CI, confidence interval.

**Table 4:** Diagnostic performance of on-site, video, and GPT-4o evaluations compared with Manikin-derived reference standards for CPR performance metrics.

	Item	Accuracy (95% CI)	Sensitivity (95% CI)	Specificity (95% CI)
Onsite vs. Manikin	Hand position/posture	70.0 (60.5–78.4)	84.3 (74.7–91.4)	25.9 (11.1–46.3)
	Compression rate	51.8 (42.1–61.4)	82.6 (68.6–92.2)	29.7 (18.9–42.4)
	Compression depth	67.3 (57.7–75.9)	81.2 (69.5–89.9)	47.8 (32.9–63.1)
	Full chest recoil	46.4 (36.8–56.1)	77.8 (62.9–88.8)	24.6 (14.8–36.9)
Video vs. Manikin	Hand position/posture	72.7 (63.4–80.8)	88.0 (79.0–94.1)	25.9 (11.1–46.3)
	Compression rate	49.1 (39.4–58.8)	82.6 (68.6–92.2)	25.0 (15.0–37.4)
	Compression depth	55.5 (45.7–64.9)	62.5 (49.5–74.3)	45.7 (30.9–61.0)
	Full chest recoil	54.5 (44.8–64.1)	77.8 (62.9–88.8)	38.5 (26.7–51.4)
GPT-4o vs. Manikin	Hand position/posture	73.6 (64.4–81.6)	94.0 (86.5–98.0)	11.1 (2.4–29.2)
	Compression rate	35.5 (26.6–45.1)	78.3 (63.6–89.1)	4.7 (1.0–13.1)
	Compression depth	55.5 (45.7–64.9)	40.6 (28.5–53.6)	76.1 (61.2–87.4)
	Full chest recoil	55.5 (45.7–64.9)	35.6 (21.9–51.2)	69.2 (56.6–80.1)

CI, confidence interval.

**Table 5:** Comparative analysis of diagnostic performance between human evaluators (On-site, Video) and GPT-4o for CPR performance metrics.

	Item	Metric	Difference, % (95% CI)	*p* Value
On-site vs. GPT-4o Evaluator	Hand position/posture	Accuracy	−3.6 (−9.8–2.5)	0.386
		Sensitivity	−9.6 (−16.0–−3.3)	0.013
		Specificity	14.8 (1.4–28.2)	0.134
	Compression rate	Accuracy	16.4 (7.1–25.6)	0.002
		Sensitivity	4.3 (−9.1–17.8)	0.752
		Specificity	25.0 (12.8–37.2)	<0.001
	Compression depth	Accuracy	11.8 (−0.7–24.3)	0.093
		Sensitivity	40.6 (25.8–55.5)	<0.001
		Specificity	−28.3 (−43.8–−12.7)	0.004
	Full chest recoil	Accuracy	−9.1 (−22.1–3.9)	0.221
		Sensitivity	42.2 (25.4–59.1)	<0.001
		Specificity	−44.6 (−57.4–−31.8)	<0.001
Video vs. GPT-4o Evaluator	Hand position/posture	Accuracy	−0.9 (−7.3–5.5)	1.000
		Sensitivity	−6.0 (−13.0–0.9)	0.182
		Specificity	14.8 (1.4–28.2)	0.134
	Compression rate	Accuracy	13.6 (4.7–22.5)	0.007
		Sensitivity	4.3 (−9.1–17.8)	0.752
		Specificity	20.3 (8.7–31.9)	0.004
	Compression depth	Accuracy	0.0 (−10.7–10.7)	1.000
		Sensitivity	21.9 (8.5–35.2)	0.006
		Specificity	−30.4 (−43.7–−17.1)	<0.001
	Full chest recoil	Accuracy	−0.9 (−13.1–11.3)	1.000
		Sensitivity	42.2 (25.4–59.1)	<0.001
		Specificity	−30.8 (−43.5–−18.0)	<0.001

## Data Availability

The raw data supporting the conclusions of this article will be made available by the authors on request.

## Supplementary Materials

The following supporting information can be downloaded at: https://www.mdpi.com/article/10.3390/jcm15124436/s1, Table S1: Full Prompt for GPT-4o-Based CPR Skills Evaluation.

## Abbreviations

The following abbreviations are used in this manuscript:

AED	Automated External Defibrillator
AI	Artificial Intelligence
BLS	Basic Life Support
CPR	Cardiopulmonary Resuscitation
FPS	frames per second
2D	two-dimensional
3D	three-dimensional
ICC	Intraclass Correlation Coefficient
VLMs	Vision–Language Models

## Appendix A. Detailed Scoring Criteria for the CPR Skills Evaluation

	**Criteria**	**Score**
Patient assessment	Check responsiveness (shoulder tapping)	1 point
	Call for emergency services (119)	1 point
	Request AED	1 point
	Check breathing	1 point
Chest compression	Correct hand position for chest compressions	1 point
	Compression rate of 100–120/min (30 compressions completed within 15–18 s)	1 point
	Compression depth of 5–6 cm	1 point
	Appropriate chest recoil (complete chest relaxation between compressions)	1 point
Defibrillation	Turn on AED immediately upon arrival	1 point
	Correct placement of AED pads	1 point
	Clear bystanders during rhythm analysis (verbal and physical action)	1 point
	Clear bystanders during shock delivery (verbal and physical action)	1 point
AED, Automated External Defibrillator.		
