# Supplementary material for: Complementary Error Patterns Between Human Evaluators and GPT-4o in Video-Based Cardiopulmonary Resuscitation Skills Assessment: Implications for Artificial Intelligence-Assisted Second Reading
Source: J Clin Med. 2026 Jun 8;15(12):4436. doi: 10.3390/jcm15124436 (PMC13301782; doi:10.3390/jcm15124436)
Supplement: Supplementary file 1 [file jcm-15-04436-s001.zip › jcm-4292261-supplementary.pdf]

Table S1. Full Prompt for GPT-4o-Based CPR Skills Evaluation

After providing cardiopulmonary resuscitation (CPR) training to laypersons, the participants performed a test according to a predefined protocol, and their performance was recorded on video. From this point onward, you are required to act as a strict evaluator of layperson CPR chest compression performance. You will be given one video and a checklist.<br><br>The scenario begins when the participant encounters a patient in cardiac arrest. The participant is expected to perform the following sequence of actions.<br>1. Check responsiveness : (While tapping the patient's shoulders) "Hello! Open your eyes! Wake up!"<br>2. Call for help : Instruct a bystander to call emergency services (119) and bring an automated external defibrillator (AED)<br>3. Check breathing for at least 5–10 seconds<br>4. Start CPR : Perform 30 chest compressions and 2 rescue breaths per cycle, for a total of 3 cycles<br>5. Use AED : Turn on the AED. Instruct bystanders to stand clear for analysis(both verbally and with actions). Instruct bystanders to stand clear for shock delivery (both verbally and with actions)<br>6. After shock, immediately resume chest compressions<br><br>Please remember the scoring guidelines that describe the evaluation criteria and scoring system for the chest compression test. Using these rules, fairly evaluate each participant's CPR performance shown in the video file. Based on the above scoring guidelines, assess the participant's chest compression performance in the video and report evidence for any point deductions. Begin the evaluation and provide both the score and the justification for deductions. Do not consider any previously uploaded video files. If you are unable to process the video, continue attempting alternative methods until successful.<br><br>The scoring criteria are as follows:<br>Check responsiveness (shoulder tapping) (1 point)<br>Call for emergency services (119) (1 point)<br>Request AED (1 point)<br>Check breathing (1 point)<br>Correct hand position for chest compressions (1 point)<br>Compression rate of 100–120/min (30 compressions completed within 15–18 seconds) (1 point)<br>Compression depth of 5–6 cm (1 point)<br>Appropriate chest recoil (complete chest relaxation between compressions) (1 point)

Turn on AED immediately upon arrival (1 point)
Correct placement of AED pads (1 point)
Clear bystanders during rhythm analysis (verbal and physical action) (1 point)
Clear bystanders during shock delivery (verbal and physical action) (1 point)
